# A Low-Cost Three-Dimensional DenseNet Neural Network for Alzheimer’s Disease Early Discovery †

**DOI:** 10.3390/s21041302

**Published:** 2021-02-11

**Authors:** Braulio Solano-Rojas, Ricardo Villalón-Fonseca

**Affiliations:** CITIC-ECCI, Universidad de Costa Rica, San José 11501, Costa Rica; ricardo.villalon@ucr.ac.cr

**Keywords:** Alzheimer’s disease, magnetic resonance imaging, optical sensors, image processing, deep learning, computer-aided detection, computer-aided diagnosis

## Abstract

Alzheimer’s disease is the most prevalent dementia among the elderly population. Early detection is critical because it can help with future planning for those potentially affected. This paper uses a three-dimensional DenseNet architecture to detect Alzheimer’s disease in magnetic resonance imaging. Our work is restricted to the use of freely available tools. We constructed a deep neural network classifier with metrics of $\bar{0.86}$ mean accuracy, $\bar{0.86}$ mean sensitivity (micro-average), $\bar{0.86}$ mean specificity (micro-average), and $\bar{0.91}$ area under the receiver operating characteristic curve (micro-average) for the task of discriminating between five different disease stages or classes. The use of tools available for free ensures the reproducibility of the study and the applicability of the classification system in developing countries.

## 1. Introduction

Alzheimer’s Disease (AD) is the most prevalent dementia among the elderly population [1]. AD is a neurodegenerative disease without a known cure. Therefore, early detection strategies have become an important research focus in the absence of an effective treatment for AD. The proper identification of AD patients allows for adequate planning for the future and the necessary modifications to living accommodations and lifestyle [1]. Some dietary changes or medications have proven to slow disease progression [2]. Additionally, knowing AD’s development can help families psychologically prepare for the future and the necessary changes in the attention that needs to be given to their family members [1].

The advancement of early AD detection has resulted in initiatives such as the Alzheimer’s Disease Neuroimaging Initiative (ADNI). ADNI is a repository of images and biomarkers of healthy and AD-affected individuals available through their website (http://adni.loni.usc.edu). ADNI originated in 2003 as a public and private endeavor, led by Dr. Michael W. Weiner. ADNI’s primary objective is to question whether medical images, other biomarkers, and clinical or neuropsychological evaluation can be united to scale AD’s progress. Using the corpus of ADNI clinical images, it is possible to develop AD early detection software tools, commonly referred to as Computer-Aided Detection (CAD) [3]. CAD assists the clinical workers by providing a support decision system to help them detect diseases.

The potential of human error is a primary reason to develop software for the early detection of AD. The process of image-based early detection by health care professionals is affected by factors such as distractions, stress, fatigue, and inherent cognitive biases regarding the disease’s specific conditions. Lee et al. [4] affirmed that about 75% of all medical mistakes were due to radiologists’ diagnostic errors. Similarly, Graber et al. [5] observed that cognitive determinants contribute to approximately 74% of diagnostic errors.

Lee et al. [4] and Graber et al. [5] considered that the most common causes of diagnostic mistakes are inadequate systems, cognitive bias, fatigue, stress, and a high workload. Consequently, the development of CAD driven by artificial intelligence using Deep Neural Networks (DNNs) is increasing. These tools show remarkable proficiency in recognizing various diseases using clinical images. For example, the work by Rajpurkar et al. [6], where DNNs classified pneumonia in chest X-rays, demonstrated their power by outperforming radiologists.

The ability of DNNs for clinical image classification enables the development of diverse DNN architectures. These can be combined into computer-aided detection systems to identify disease progression and detection such as for AD. Hence, DNNs are relevant and a staple in clinical image analysis and diagnostics. A type of DNN, the Convolutional Neural Network (CNN), inspired by the inner workings of living organisms’ visual cortex, is a recognized architecture for image analysis and is currently used in the field of computer vision. The scope of use of CNNs involves self-driving cars, drones, robotics, sports and recreation, intelligent surveillance and monitoring, and health and medicine [7].

In [8], we created a three-dimensional CNN architecture, specifically DenseNet-121, trained using the ADNI MRI image dataset. We measured the accuracy of detecting Alzheimer’s disease with the implemented DNN architecture. This manuscript extends our previous work by further explaining our data preprocessing with the inclusion of figures. Additionally, we reevaluate our classifier using 16 runs with randomized data partitions instead of just one run. Finally, we include and discuss attention maps to convey what the constructed neural network perceives. Attention maps provide an interpretation of neural networks, often criticized for being black boxes that do not explain their output. Attention maps also help to analyze the neural network developed.

As in [8], we kept our low-cost approach. Our goal is to provide a technological artifact that can be used across many health care services to benefit people, particularly for developing countries with difficulties accessing specialized computing platforms.

This paper first provides some background definitions in Section 2 to support our work. In Section 3, we describe previous work in more detail. Then, in Section 4, we provide the methodology employed to realize this work. We present in Section 5 the results of the design choice. We discuss those results in Section 6. We finalize our discussion with concluding remarks and future work in Section 7.

## 2. Background

We begin with a brief review of the medical glossary used to provide a context for our research. We present distinct clinical grades of disease that we desire to classify. Later, we report two types of medical imaging used in the discovery and diagnosis of AD.

### 2.1. Clinical Disease Stages

Cognitively normal, significant memory concern, and mild cognitive impairment are the distinct stages before AD’s clinical diagnosis.

#### 2.1.1. Cognitively Normal

In the ADNI study, the control subjects are Cognitively Normal (CN) patients. They exhibit no symptoms of depression, mild cognitive impairment, or dementia. They are aging in a healthy manner [9].

#### 2.1.2. Significant Memory Concern

Significant Memory Concern (SMC) is reported by the patient. SMC is quantified using the Cognitive Change Index and a Clinical Dementia Rating of zero. Subjective memory concerns are correlated with a greater possibility of progression, thereby lessening risk stratification amid normal controls and addressing the gap within healthy elderly controls and mild cognitive impairment. Nevertheless, SMC patients score inside the typical range for cognition [9].

#### 2.1.3. Mild Cognitive Impairment

Mild Cognitive Impairment (MCI) patients have a subjective memory concern, either self-reported or through a clinician or informant. Nonetheless, everyday living activities are essentially maintained; there are no meaningful impairment levels in different cognitive domains, and no symptoms of dementia exist. Levels of MCI (early or late) are defined using the Wechsler Memory Scale Logical Memory II [9].

#### 2.1.4. Alzheimer’s Disease

AD is the most common form of dementia. Dementia is a generic name for memory and other cognitive capabilities’ decline. Dementia is severe enough to affect everyday life. AD is a gradual disease, where dementia signs progressively worsen beyond several years. Individuals suffer the loss of the capacity to converse and react to their surroundings. Current medicines cannot stop the disease from advancing. Medications can temporarily delay the worsening of dementia signs and enhance life quality for those with AD and their caregivers [1].

Because we intend to assess if those stages are identified from medical imaging, expressly magnetic resonance imaging, we proceed to describe two medical imaging methods.

### 2.2. Medical Imaging

Medical imaging is the procedure and technique of producing visual representations of the human body’s inside for clinical examination and medical intervention. We present two types of medical imaging, although we are particularly interested in Magnetic Resonance Imaging (MRI) as input for DNNs. We also introduce Positron Emission Tomography (PET) because it is sometimes a modality that accompanies MRI. Next, we explain what MRI and PET are.

#### 2.2.1. Magnetic Resonance Imaging

MRI is a non-invasive imaging technique that generates a detailed volumetric anatomical visualization without harmful radiation of human tissues. It is applied frequently for treatment monitoring and disease detection and diagnosis. It is based on advanced technology that excites and detects the difference in the direction of the rotational axis of protons located in the water that constitutes living tissues [10].

#### 2.2.2. Positron Emission Tomography

PET scans employ radiopharmaceuticals to produce volumetric images. This type of scan generates small particles named positrons. A positron is a particle with approximately equal mass to an electron, but oppositely charged. Positrons respond to electrons in the body, and when these two particles unite, they annihilate each other. This annihilation briefly generates an amount of energy in the configuration of two photons that shoot off in opposite directions. These photons are measured by the detectors in the PET scanner, and this information is employed to produce images of internal organs [11].

## 3. Previous Work

Our literature review focuses on the current state of deep learning for AD detection and how much further this field can be improved through convolutional neural networks. We employed IEEE (https://ieeexplore.ieee.org/) as the reference for artificial neural networks because, based on the journal rankings (https://www.scimagojr.com/) for the subject of artificial intelligence, it is the highest-ranked in both the SCImago Journal Rank (SJR) and H-Index. We used Google Scholar and Duck Duck Go for other publications.

In the IEEE Digital Library, we employed the search string “deep AND learning AND alzheimer AND mri” to identify the amount of cases where the classification and detection of AD used convolutional neural networks. We ran the query from the year 2016 to the year 2019. The search resulted in 81 documents recovered from the IEEE Digital Library, including early access articles, journals, and conferences. As of 2020, the query result provided an additional 39 results. The new results indicate that the research field of Alzheimer’s disease detection with CNNs remains relevant.

Previously, in [8], we selected by title, and if the title was cryptic, we also used the abstract. We focused on the implementation of convolutional neural networks. The selection resulted in 32 articles. We focused on literature that covered convolutional deep learning classifiers’ study to identify AD in MRI and other modalities. We restricted the review to supervised learning, thus omitting architectures such as convolutional autoencoders. We also screened the 39 new articles and found 10 additional articles of interest. For the collected studies that met our selection criteria, we extracted the following information: (1) publication year, (2) architecture of the neural network, (3) if the MRI images were processed, (4) the modalities (number of inputs), (5) the number of classes used in the classifier, (6) accuracy, (7) sensitivity, (8) specificity, and finally, (9) the Area Under the Receiver Operating Characteristic curve (AUROC).

As stated in [8], we identified a concern when analyzing the collected papers and their data. Approximately 50% of papers report accuracy, but did not report sensitivity, specificity, or AUROC. It appears that there is a competition to achieve higher accuracy, although this measure is misleading. A classifier may report a high accuracy and still possess a low capability of correct prediction.

We concluded that the various research efforts are too distinct for an adequate comparison and contrast based on the collected information. We noticed the avoidance of multiclass classification. The most commonly reported classifier systems were binary, which usually results in higher accuracy. By increasing the number of classes, it is not uncommon to see the classifiers’ performance decrease. However, multiclass models are more informative than binary classifiers. In our search, the multiclass classifiers identified had a maximum of four classes.

When looking for specific architectures, we noticed that there were not many entries that used the DenseNet architecture. We did encounter papers with other varieties of residual neural networks: three-dimensional VoxResNet [12], ResNet [13,14,15], and three-dimensional ResNet [16]. Only three documents used DenseNets, of which two [17,18] were three-dimensional, but with depthless DenseNets, and one [19] used deep DenseNets, but two-dimensional. The collected items’ quantitative analysis did not generate a great contribution due to these defects. However, in the studies’ review, we found documents of remarkable quality like [20]. We also judged that some of the manuscripts collected were not easily repeatable, for example [13].

Finally, when looking at the 10 additional papers acquired from the IEEE Digital Library, four used three-dimensional deep learning [21,22,23,24]. We believe that, as mentioned by [21], three-dimensional architectures are being initiated. We consider that this kind of architecture is growing in use. We also continued to find research efforts that only report high accuracy, but not other metrics [24,25,26,27,28]. Thus, the paradigm of obtaining the highest possible accuracy in classification remains. Last, we found it interesting that [21] reported MRI as a better modality than PET.

In contrast to previous research, we explored the process of constructing a multiclass neural network classifier system using tools available for free. Additionally, we report multiple performance metrics such as sensitivity, specificity, AUROC, and the model’s accuracy, in contrast to focusing on reporting only the classifier’s accuracy. Lastly, we are committed to reproducibility, describing our methods and parameters used in the next section.

## 4. Materials and Methods

This section describes how we collected the ADNI data and how we preprocessed them. Next, we introduce our development and how we created, using the Google Colaboratory tool, an Alzheimer’s prediction model to achieve the goal of estimating the accuracy of the detection of Alzheimer’s disease employing a three-dimensional DenseNet-121.

### 4.1. Data Retrieval

We used the beta advanced search functionality of ADNI with the following criteria: In Projects, we checked ADNI. In Research Group, we checked MCI, EMCI, AD, SMC, and CN. In Modality, we checked MRI. We only selected MRI and excluded PET based on an economic constraint criterion. PET requires radiopharmaceuticals. Therefore, it is more common to find MRI being used in economically restricted circumstances.

Regarding other search options, for Image Description, we used the string MPRAGE; for Acquisition Plane, we selected SAGITTAL; and finally, in Weighting, we selected T1. We kept the remaining search fields with default parameters. With these parameters, we collected 5556 magnetic resonance images with the demographics in Table 1.

The MRI acquired from ADNI is in the Digital Imaging and Communication On Medicine (DICOM) format. The images are a zipped archive of 55.5 GB, and the uncompressed files hold 138 GB. We decrease that quantity with image preprocessing, and we describe how and why in the following section.

### 4.2. Data Transformation

**Figure 1:**
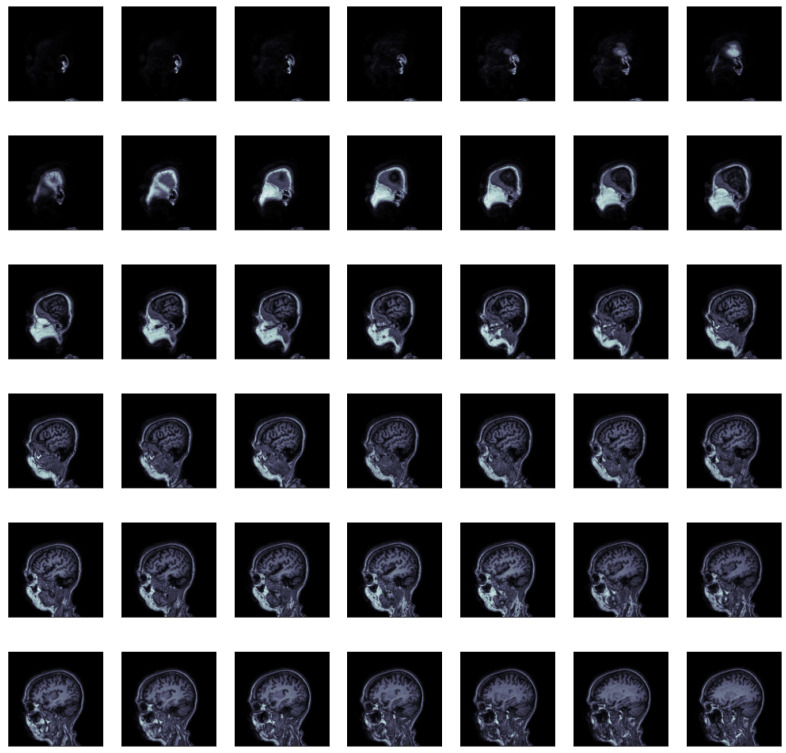
Volumetric data from a subject’s MRI (extracted slice).

MRI data comprise groups of slices. We present in Figure 1 an extract of the MRI slides of a subject. The extract is not complete. We only show 42 slides out of 170. Each slice is an image, and the combination of images forms the MRI. All images or slices are a pixel matrix. Every slice has an associated spatial thickness because they reproduce reality. Furthermore, all pixels in each slice have a spacing, that is the space they represent. Consequently, the data are volumetric or rectangular cuboids.

Considering the volumetric nature of the data, we applied the next transformations to it. First, we transformed all volumetric pixels (voxels) to a spacing of 1×1×1 millimeters. This transformation may add or delete slices or slice voxels. Next, we turned each slice into 256×256 voxels as follows. Some slices are not square. When they are not, we filled them in with black voxels. After they were equilateral, if they were not 256×256, we changed them to that dimension employing interpolation. Likewise, we made the cuboids have 256 slices utilizing interpolation. Cubes of 256×256×256 resulted from this. From these cubes, we created a cut from Slice 40 to Slice 214, from Row 50 to Row 199, and from Column 40 to Column 239, to keep only the area with brain tissue. This cut allowed discarding the black voxels’ edges and preserving the useful internal information (the brain). Because we made all the MRIs the same size, we assumed that the cut conserved to the brain. We did not apply methods like segmentation (splitting the brain using pattern recognition). Of those cuboids, we employed only half of the slices and half of every slice’s rows and columns by dropping one in between for all. The latter diminished the dimensionality of the problem and the volume of the images considerably. Finally, we normalized the voxels’ values to an interval of −1.0 to 1.0.

**Figure 2:**
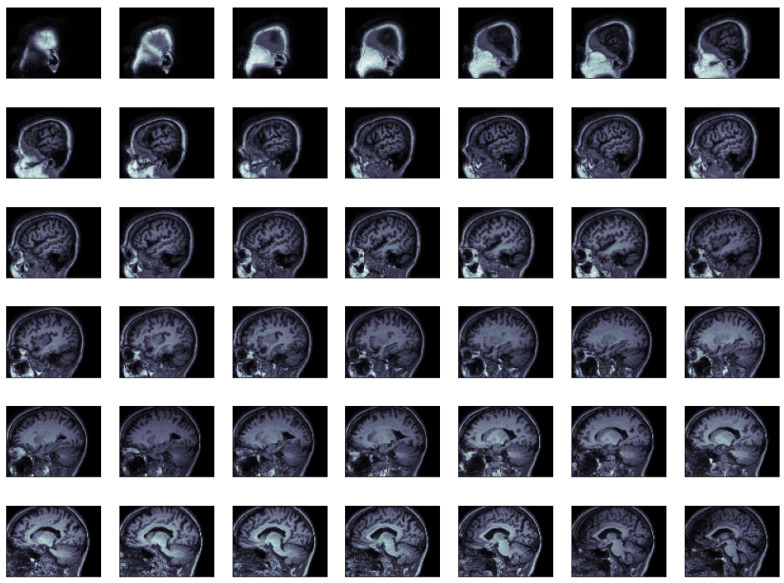
Processed volumetric data from a subject’s MRI (extract of the processed slices).

In Figure 2, we show an extract of a subject’s processed slices to illustrate these transforms. It is the same subject as in Figure 1. The transform was still volumetric data. The extract is not complete because we only show 42 slices out of 87. As can be seen from the slices, we cut rectangles from the original slices, and these rectangles are compressed, but still show the brain patterns.

Data transformation can be done both previously or online. We implemented both. Nevertheless, to sustain a low-cost goal, we employed a script to apply the transformation previously to the job of neural network training. We used the already transformed MRI. Preprocessing beforehand may be achieved on a laptop or desktop computer. Although that would take hours, it is not a process that would demand more than a day on contemporary hardware.

After data transformation, the images were only 13.5 GB. We decreased the size of the images by approximately more than ten times. This compression helped minimize our development’s neural network storage needs and training time. The next section explains that development.

### 4.3. Model Construction

We selected a convolutional DNN of the DenseNet Bottleneck-Compressed architecture because this kind of neural network structure has an exemplary performance with fewer training parameters [29], hence using fewer resources. We based our implementation on Hara et al. [30]. They based their implementation on the two-dimensional programming available in PyTorch. However, their implementation is not general purpose, but specific. It implements video and incorporates sample_size and sample_duration as variables related to video sample size and duration. We generalized their implementation, and now, it operates with all types of cuboids. Additionally, we added a channel parameter because the implementation only considered three channels (usually red, green, and blue colors), yet the MRI is monochromatic.

With this new programming, we set the training process of the neural network with the next parameters.

Training Of the data retrieved from ADNI, we used 75% as the training dataset. The training dataset was taken randomly from the full data set.
Batch size We selected a batch size of 5 MRI images for training based on the experimental results of [20].
Testing The remaining 25% of the data was the testing dataset.
Channels The images were monochromatic; therefore, we set channels as 1 in the constructor of the neural network.
Classes At first, we set the number of classes to 6 in the neural network. Nevertheless, we chose to drop the SMC class because we believe it is a noisy subjective class. We finally set the number of classes to 5.
Dropout We used a dropout rate of 0.7 based on the investigation of [20] because it reduces overfitting.
Loss For the loss function, we employed cross-entropy. It is useful in classification problems with more than two classes.
Optimizer We used Stochastic Gradient Descent (SGD) as the optimizer. SGD is used in the event of unbalanced data like in our dataset.
Learning We employed a learning rate parameter of 0.1 in the SGD optimizer. At Epoch 80, we dropped the learning rate by 0.1 (we started at Epoch 0). That drop diminished the learning rate to a value of 0.01.
Momentum We used a standard momentum of 0.9 because an SGD optimizer with momentum typically attains flatter local minima.
Epochs We established the maximum number of epochs as 80 (in a run) because we used the Google Colaboratory platform. Due to platform’s limitations, it was impossible to exceed 90 and reach the desired 110 epochs because it disconnected us. As a result, we saved the model at Epoch 80. Because of Google Colaboratory’s constraints, explained next, we needed to wait 12 h before continuing the training. Then, we restarted training from the 80th epoch until Epoch 110.

According to other authors, the free-of-charge resources of Google Colaboratory “are far from enough to solve demanding real-world problems and are not scalable” [31]. However, we used Google Colaboratory to access the Graphics Processing Unit (GPU) computing. With the mentioned parameters, we pushed the Google Colaboratory platform’s boundaries to deliver a state-of-the-art DNN. This choice has constraints and consequences. As described in [31], the free GPU backend can be used for a maximum of 12 h. However, we believe that we had less than the allocated 12 h on some occasions, with approximately 10 h of use. The subsequent disconnection from Google Colaboratory occurred, dropping the virtual machine with its corresponding GPU. When the user immediately reconnects, the platform provides a new machine. However, it grants only 3 h of GPU backend. After the 3 h, it is impossible to use a GPU backend for a defined amount of time (12 h). To overcome the 3 h restriction, the user has to wait 12 h after the first 12 h run, as we described in the parameters. Such constraints limit the process of training and testing to a maximum of 12 h, which depending on the task, might be unfeasible.

Google Colaboratory’s constraints have other consequences. For example, it is common to validate or test neural networks throughout training, thus analyzing the neural network’s accuracy and loss across all epochs. Nonetheless, to decrease the computation time, validation or testing of the DNN was only executed at the end of the training. We decided this since a validation cycle of 25% of the data needed around 2 or 3 min, requiring 1 h or more every 30 epochs. We accepted this trade-off. It is possible to save intermediate neural network states and evaluate those states after completing the training. However, this decision also signifies that methods like early stopping can not be applied. Finally, Google Colaboratory also has disk size limitations that we overcame with our data transformation.

Based on the specified configuration parameters and considering all the constraints associated with Google Colaboratory, we were able to achieve the results discussed in the following section.

## 5. Results

**Figure 3:**
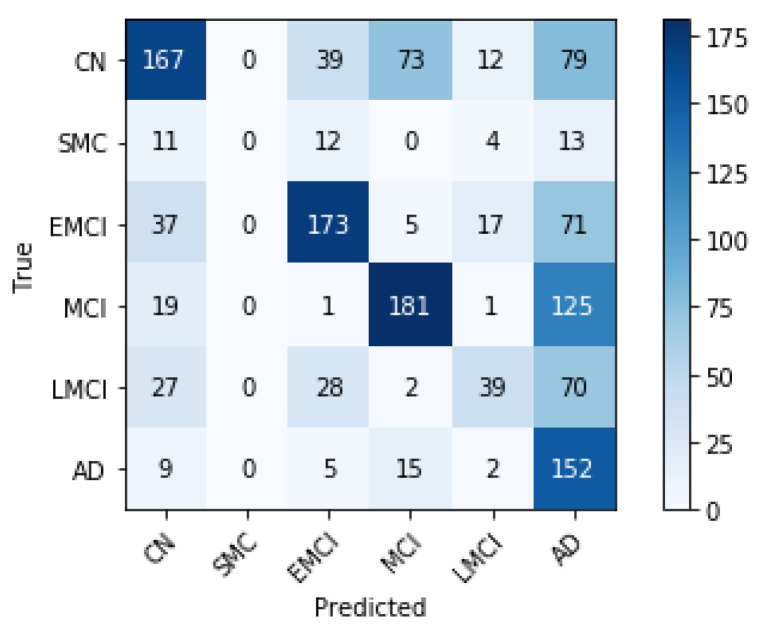
Confusion matrix with the SMC class at 50 epochs.

With the mentioned limitations, we progressively achieved our results by increasing the epochs. We first trained with six classes to 50 epochs. Figure 3 shows that, after training to 50 epochs, the SMC class is not properly discriminated. The column of the predicted SMC class is filled with zeros. Remarkably, the rest of the classes have an adequate level of correct classification. We decided to remove the SMC class from the complete dataset. The deletion of this class decreased the complete dataset from 5556 MRIs to 5370 MRIs.

**Figure 4:**
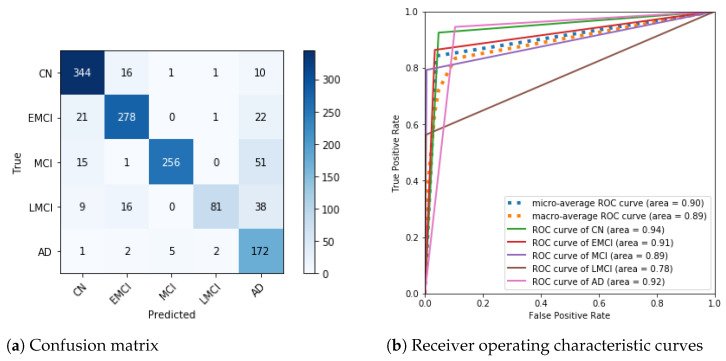
Evaluation plots of DenseNet-121 at 80 epochs.

After removing the SMC class and training to 80 epochs, the neural network for the remaining classes showed good classification metrics. These results are shown in Figure 4a,b. The confusion matrix (Figure 4a) shows how most values are in the diagonal, with fewer incorrect predictions, especially compared to the confusion matrix that included the SMC class (Figure 3). The classifier’s predictive power can be seen in Figure 4b, showing its potential for each class and all classes together.

The model was improved further, although it might be considered, based on Figure 4a, that our classifier already had a good performance. To further improve our model, we restarted the training from Epoch 80 to 110 epochs. The last predictive model had the metrics in Table 2 (page 11).

**Figure 5:**
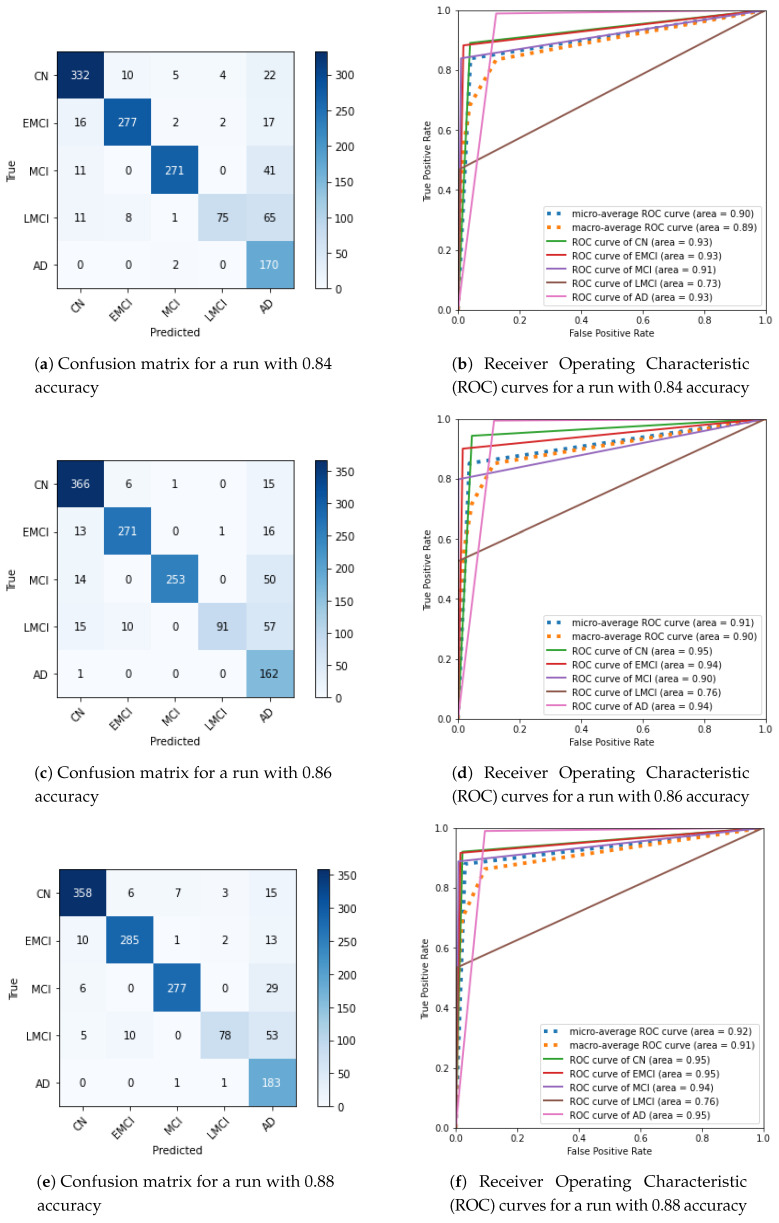
Evaluation plots of DenseNet-121 at 110 epochs.

To produce evaluation metrics, we divided the entire dataset into four parts: 1342, 1342, 1343, and 1343 images. We then undertook a test run with each of these partitions. We did this four times, which resulted in 16 runs in total. We report the mean, minimum, and maximum values for specificity, sensitivity, f1-score, and support. For support, we also report the standard deviation because the classes were not balanced. Additionally, we show sample confusion matrices and plot the area under the Receiver Operating Characteristic (ROC) curves for the worse, median, and best accuracies. This is shown in Figure 5. The reported accuracies are: 0.84, 0.86, and 0.88 (respectively). We discuss the predictive performance and metrics in the next section.

## 6. Discussion

Our first finding was that significant memory concern was noisy for training. We inferred this from the results in Figure 3. This property can be because the class is biased and is likely formed of at least two classes: people who will not develop the disease and people who will. Besides, people who will develop the disease may possess distinct progression levels, being, in turn, a class formed of different classes. The size of the SMC class may be another reason why it is problematic. It is the smallest cohort by far. These may be the causes that make it difficult to classify. We decided to remove this class.

We obtained the confusion matrix in Figure 4a after dropping the SMC class and training to 80 epochs. In the matrix, we may see that the incorrect predictions are principally pessimistic. That is, there are more errors above the diagonal than under it. This arrangement of errors above the diagonal means that the classifier makes errors that predict the upper disease stages. This kind of error is clearly in favor of patients because a false positive is better than a false negative in diagnosing diseases. Figure 4b also shows that the area under each curve tends to 1.0; which demonstrates the classifier’s diagnostic ability. Notwithstanding the faults of our classifier, it is a valuable classifier. Next, we still improve this model.

As we introduced in Section 5, we trained our final model up to 110 epochs. We extended what we previously undertook in [8]. We evaluated the training to 110 epochs with more rigor. We carried out 16 test runs, as described in Section 5.

We also created new confusion matrices and ROC curve plots (Figure 5). When analyzing the newly obtained confusion matrices, we noticed a possible pattern (Figure 5a,c,e). In the AD prediction column, we see an ascending number of false AD as there is more disease progression. There is possibly a feature that is recognized as AD as the disease progresses. Healthy people possess this feature. These false AD predictions require further investigation. However, without investigation, we believe that the solution would be the brain segmentation we propose in Section 6.1.

As mentioned in Section 3, accuracy can be a misleading metric. The confusion matrix in Figure 5c has better accuracy than the confusion matrix in Figure 5a; however, it has more elements under the diagonal. It is preferable to have more elements above the diagonal in the confusion matrix in health care and clinical diagnostics. The opposite is optimistic, and in diagnostic terms, that can be a risk. A false negative can be counterproductive, as a healthy person is misdiagnosed, with deleterious consequences. It is common in health care clinical diagnostics to prefer a pessimistic approach to an optimistic one. We commonly opt for false positives over false negatives in that context.

As the ability to differentiate between most classes improved, we noticed a decrease in the late mild cognitive impairment class’s performance metrics. We inferred that we advanced towards a local minimum solution that increased the other classes’ predictive power, but departed from the LMCI class’s accurate prediction. We believe that the need for class balance in the dataset is a possible cause of this observation. The LMCI is the class with the lowest number of instances (after the removal of SMC). The pitfall of class imbalance and the number of instances could potentially be addressed with data augmentation, as proposed in [32]. However, data augmentation would introduce a performance constraint and reduce the highest amount of epochs we can use during training. Data augmentation is a trade-off we decided not to make. Additional inspection of the LMCI prediction showed that many of the misclassified instances were labeled as AD. We can consider this a pessimistic behavior, and therefore, it was tolerated. Consequently, we accepted the final metrics and the compromise of not balancing the data through data augmentation because of the limitations imposed by Google Colaboratory.

We present additional classification metrics of this final model in Table 2. In that table, the lowest values are for AD specificity (precision) and LMCI sensitivity (recall). We could also include the sensitivity (recall) of MCI in the low numbers. The MCI 85% sensitivity (recall) will miss 25% of MCI patients; however, they will be classified pessimistically in an advanced disease stage. The AD class’s low specificity is acceptable because it reaches almost 100% sensitivity (recall). Therefore, our approach is a useful diagnostic method for AD, although not suitable for screening. Screening methods must possess high specificity metrics, whereas diagnostic approaches should have high sensitivity scores. An explanation of why the specificity metric is low is the number of multiple instances misclassified as AD. Nevertheless, false positives are prophylactic and reduce future risks in a diagnostic task.

Our results also show that the AUROC and accuracy values do not contradict each other. That consistency supports that our obtained results are consequent with each other.

We constructed our models considering compromises due to the use of freely available tools. We developed methodologies that attempted to counter the limitations and achieve the best possible classification for diagnosis (high sensitivity). Our model’s predictive ability has values of $\bar{0.86}$ mean accuracy, $\bar{0.86}$ mean specificity (micro), and $\bar{0.86}$ mean sensitivity (micro), which overall is considered acceptable. These metrics emphasize that our classifier can satisfactorily achieve the corresponding task of discriminating between the classes, even with our limitations. We decided to report final micro-average numbers instead of the macro-average. In a multiclass classification structure, the micro-average is preferred when there is a class imbalance. Still, the macro-average and the weighted average are higher. Finally, we only report the variance for support because, for the other columns, the variance is close to zero.

In the next section, we provide a study of what the neural network perceives.

### 6.1. Attention Maps

**Figure 6:**
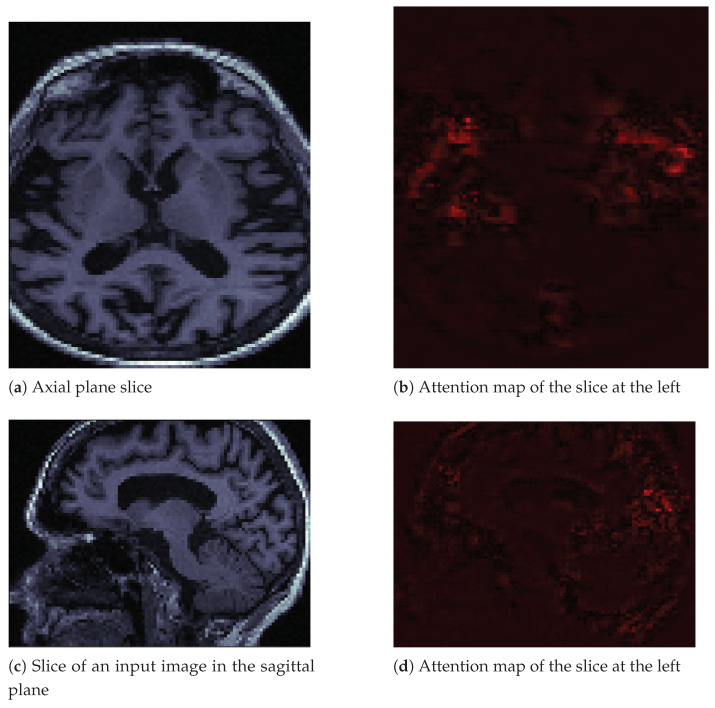
Input image slices and their attention maps from an MRI of a patient with AD.

Attention maps are visual tools that explain deep convolutional neural networks [33]. We used the M3d-Cam tool [34] with the Guided Gradient-weighted Class Activation Mapping (Grad-CAM) algorithm. We generated new images through M3d-CAM, and with these images, we could interpret what the neural network was emphasizing to make a decision. For example, in Figure 6a, we can see an image in the axial plane of a person with Alzheimer’s disease. We extracted only one slice of the cuboid. To its right, in Figure 6b, we can see that the neural network focuses its attention on brain atrophy. We extracted the same slice number from the attention map cuboid. In Figure 6c,d, we see the same patient and the same features in the sagittal plane. We also only extracted one slice of that plane.

In the images in Figure 6, we can see how brain atrophy areas appear more prominent. We noticed this behavior in other images for which we produced attention maps. The behavior of the network was as expected in terms of identifying the regions of importance. However, we also noticed that sometimes the neural network gave importance to bony areas of the skull or even to areas outside the skull. In other words, the attention maps seemed to have a potential function as debugging tools. Besides, they allowed us to propose solutions. We believe that we must improve the preprocessing of images by removing the skull using a mask, as the skull is a potential source of noise. This background noise could explain some of the classifier’s inaccurate results, as observed in the confusion matrices. The neural network may be learning uninformative features or patterns as a result of excess inputs. We could improve the results and reduce the inaccurate results by providing the CNN with only brain images, thus removing potential artifacts associated with the skull.

**Figure 7:**
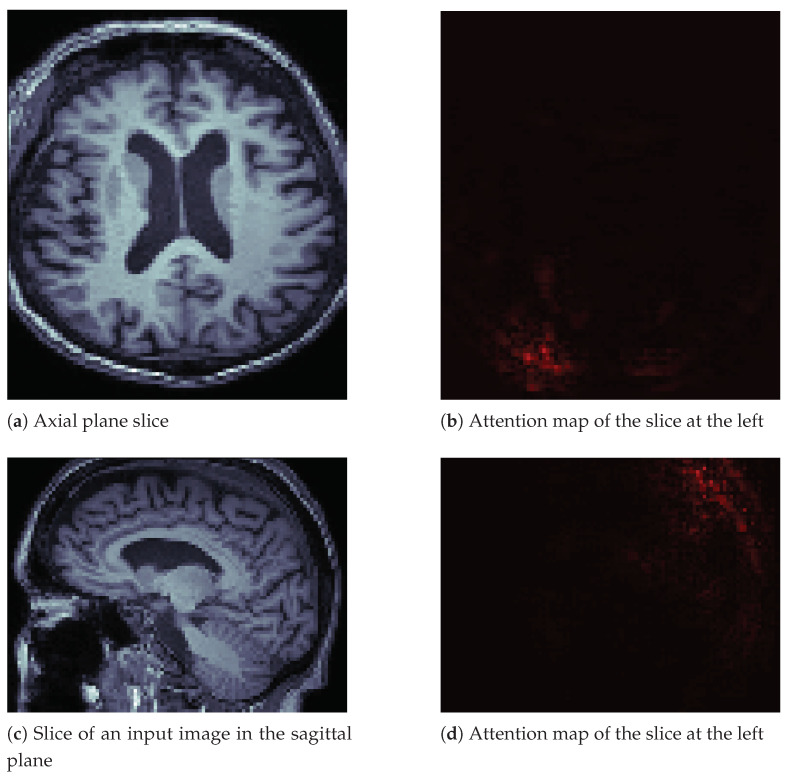
Image slices and their attention maps from an MRI of a healthy person.

Additionally, we include the attention map for a non-affected individual in Figure 7 on page 14. The difference from the image in Figure 6 is that this image seems to generate less attention from the neural network. We observed the same behavior in other images of healthy people.

The M3d-CAM tool allowed us to better study the resulting neural network and generate new hypotheses to improve our current work. Furthermore, by incorporating the attention maps, we added a layer to our work that widens what we can achieve with our current and future classification systems. As an example, we now consider the use of brain segmentation. It is essential to include more visualization tools for neural networks that would allow debugging and understanding these architectures in depth.

## 7. Conclusions

We explored a low-cost way to create a deep artificial convolutional neural network architecture for AD detection that proved its effective performance in this work. Our classification system has performance metrics of 0.86 mean accuracy, 0.86 mean sensitivity (micro-average), 0.86 mean specificity (micro-average), and 0.91 area under the receiver operating characteristic curve (micro-average).

The constructed classifier is a valuable and potentially viable option for developing countries’ diagnostic systems if we add a user interface and interpretation to the model. The model presents high individual sensitivities (recall), a required staple for computer-assisted diagnostic systems. We can use such a tool in remote medicine scenarios and deployment for AD early detection. We may base a possible CAD on Chester’s open implementation [35], a computerized disease prediction system for chest X-rays presented through the web. With the advent of recent tools such as TensorFlow.js and ONNX, we can transform models trained using PyTorch to work in a browser, and we can use WebGL for display [35]. This interface would permit prediction and also add explanation or interpretation through attention maps or heat maps. One hospital in Costa Rica has shown interest in such a tool.

By implementing attention maps, we discerned a potential focus for future improvement. We identified noisy features that could be the cause of inaccuracies. We could remove the noise by employing image processing. Using a mask, we would cut non-brain pixels. We do not attribute errors to the freely available tools used.

Additionally, we want to improve our model without using supervised learning. We will use reinforcement learning. We also will not use gradient methods for training, that is to say gradient descent and backpropagation. We will combine reinforcement learning with evolutionary computing.

We also want to perform a series of experiments. One possible experiment we could carry is the comparison of different architectures as, for instance, DenseNet-169, DenseNet-201, ResNet-18, ResNet-34, ResNet-50, ResNet-101, ResNet-152, and ResNeXt-101. We could consider every architecture as a treatment for the data and perform an analysis of the variances for comparison. In that same line, we also want to compare against human professionals in the health sector. For instance, works such as [6] compared against humans.

Another possibility of experimentation consists of mixing different data sources. For example, we could use the entire ADNI tagged data set for training only, and we could use other data sources for validation. Among the possible data sources for validation are our local health centers or Open Access Series of Imaging Studies (OASIS) [36].

Finally, regarding the transparency and reproducibility of academic work, we contribute the source code of our DNN at [37]. The produced neural network is available for download at [38].

## Figures and Tables

**Table 1:** Demographics of the images.

Subject Age		Subject Cohort		Subject Sex	
50–75 years	2811	Cognitive Normal	1520	Male	2956
75–100 years	2745	Significant Memory Concern	186	Female	2599
		Early Mild Cognitive Impairment	1222	Unknown	1
		Mild Cognitive Impairment	1274		
		Late Mild Cognitive Impairment	636		
		Alzheimer’s Disease	718		

**Table 2:** Evaluation metrics of the obtained DNN at 110 epochs.

	Specificity (Precision)	Sensitivity (Recall)	f1-Score	Support
Cognitive Normal	$\bar{0.92}\;\left[0.89,0.94\right]$	$\bar{0.93}\;\left[0.89,0.95\right]$	$\bar{0.92}\;\left[0.89,0.94\right]$	$\bar{380}\;\left[350,411\right]\;\sigma^{2}=17.13$
Early Mild Cognitive Impairment	$\bar{0.94}\;\left[0.92,0.95\right]$	$\bar{0.90}\;\left[0.87,0.92\right]$	$\bar{0.92}\;\left[0.90,0.93\right]$	$\bar{305.5}\;\left[290,322\right]\;\sigma^{2}=10.27$
Mild Cognitive Impairment	$\bar{0.98}\;\left[0.96,1.00\right]$	$\bar{0.85}\;\left[0.80,0.89\right]$	$\bar{0.91}\;\left[0.89,0.93\right]$	$\bar{318.5}\;\left[301,343\right]\;\sigma^{2}=10.27$
Late Mild Cognitive Impairment	$\bar{0.94}\;\left[0.90,0.99\right]$	$\bar{0.51}\;\left[0.42,0.55\right]$	$\bar{0.66}\;\left[0.58,0.69\right]$	$\bar{159}\;\left[145,182\right]\;\sigma^{2}=10.31$
Alzheimer’s Disease	$\bar{0.59}\;\left[0.52,0.63\right]$	$\bar{0.99}\;\left[0.98,0.99\right]$	$\bar{0.73}\;\left[0.68,0.77\right]$	$\bar{179.5}\;\left[163,201\right]\;\sigma^{2}=9.87$
Macro average	$\bar{0.87}\;\left[0.85,0.88\right]$	$\bar{0.83}\;\left[0.81,0.85\right]$	$\bar{0.83}\;\left[0.80,0.85\right]$	$\bar{1342.5}\;\left[1342,1343\right]\;\sigma^{2}=0.5$
Weighted average	$\bar{0.89}\;\left[0.88,0.90\right]$	$\bar{0.86}\;\left[0.84,0.88\right]$	$\bar{0.86}\;\left[0.84,0.88\right]$	$\bar{1342.5}\;\left[1342,1343\right]\;\sigma^{2}=0.5$
	Accuracy		$\bar{0.86}\;\left[0.84,0.88\right]$	
	Micro specificity (precision)		$\bar{0.86}\;\left[0.84,0.88\right]$	
	Micro sensitivity (recall)		$\bar{0.86}\;\left[0.84,0.88\right]$	

## Data Availability

The dataset used is available at http://adni.loni.usc.edu.

## Abbreviations

The following abbreviations are used in this manuscript:

AD	Alzheimer’s Disease
ADNI	Alzheimer’s Disease Neuroimaging Initiative
AUROC	Area Under the Receiver Operating Characteristic curve
CAD	Computer-Aided Diagnosis
CN	Cognitively Normal
CNN	Convolutional Neural Networks
DICOM	Digital Imaging and Communication On Medicine
EMCI	Early Mild Cognitive Impairment
GPU	Graphics Processing Unit
Grad-CAM	Gradient-weighted Class Activation Mapping
LMCI	Late Mild Cognitive Impairment
MCI	Mild Cognitive Impairment
MPRAGE	Magnetization-Prepared Rapid Acquisition with Gradient Echo
PET	Positron Emission Tomography
ROC	Receiver Operating Characteristics
SGD	Stochastic Gradient Descent
SJR	SCImago Journal Rank
SMC	Significant Memory Concern
